# Pericardial Effusion and Progressive Bilateral Effusion as Rare Presentations of Idiopathic Hypereosinophilic Syndrome

**DOI:** 10.7759/cureus.44495

**Published:** 2023-08-31

**Authors:** Shunsuke Soma

**Affiliations:** 1 Emergency and Critical Care Center, Aomori Prefectural Central Hospital, Aomori, JPN

**Keywords:** eosinophilic myocarditis, eosinophilic disease, pleural fluid, cardiac tamponade, hypereosinophilic syndrome

## Abstract

Hypereosinophilic syndrome (HES) is a rare disease with peripheral blood eosinophils >1500/µL and end-organ damage. We encountered a case of idiopathic HES in a woman in her 60s who presented with dyspnea due to cardiac effusion and bilateral pleural effusions. At first, the patient did not have eosinophilia in the peripheral blood, and the presence of serum pericardial fluid and pleural effusion led to suspicion of carcinomatous pericarditis and pleurisy. One month later after onset, eosinophilia in the peripheral blood was observed, and HES was suspected for the first time. However, inflammatory cell infiltration by eosinophils has been observed in the pleural fluid specimen before eosinophilia in the peripheral blood. Prednisolone was administered, and the pleural effusion and respiratory failure quickly abated. This case provided an educational illustration of a unique manifestation of cardiac tamponade and HES, characterized by the absence of peripheral blood eosinophilia at the initial presentation.

## Introduction

Hypereosinophilic syndrome (HES) is a rare disease marked by an excessive number of eosinophils in the peripheral blood and the infiltration of these cells into various tissues. The disorder is diagnosed when the absolute eosinophil count exceeds 1500/µL on two or more occasions, lasting over a month and resulting in organ damage [1]. The symptoms of tissue infiltration by eosinophils vary widely but commonly affect the skin, lung, and gastrointestinal tract [2]. Although cardiac involvement as a first symptom is infrequent, it is present throughout the disease in approximately 40-50% of patients [3] and accounts for the majority of fatalities associated with HES. In general, cardiac lesions in HES are classified into three stages. The acute necrosis stage is characterized by infiltration of eosinophils into the myocardium, resulting in myocardial damage; the thrombogenic stage, in which thrombus forms in the damaged endocardium, resulting in various thromboembolic events; and the fibrotic stage, in which thrombus is replaced by fibrosis and heart failure and valve disease appear [4]. These are diagnosed by elevated myocardial troponin and echocardiography in patients with hypereosinophilia. It is important to control the eosinophilic infiltrate at an early stage to prevent thromboembolism, heart failure, and valvular disease. Therefore, early detection and timely intervention of cardiac lesions are crucial in HES management.

This case report of an idiopathic HES who exhibited pericardial effusion and worsening respiratory failure caused by bilateral pleural effusions. The patient had an atypical presentation with cardiac involvement and an even more unusual course with a delayed appearance of hypereosinophilia.

## Case presentation

**Figure 1:**
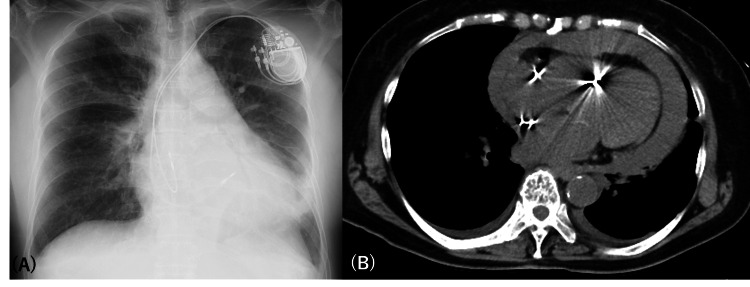
(A) Chest X-rays at the first visit to the previous physician. (B) The axial view of CT of the chest showed moderate pericardial effusions and slightly bilateral pleural effusions at the first visit to the previous physician

A female in her 60s visited her primary physician in the emergency department with symptoms of nausea, vomiting, and loss of appetite that persisted for several days. Past medical history was significant for previous radiation therapy for Hodgkin lymphoma 30 years ago, hypothyroidism, and permanent chamber pacemaker due to syncope caused by sinusoidal failure syndrome. A computed tomography (CT) revealed the presence of pericardial effusion (Figure 1A-1B).

She had normal vital signs, such as blood pressure of 105/45 mmHg and a pulse rate of 99 beats per minute, with a saturation level of 95% on ventricular air. According to vital signs, her hemodynamic state was preserved, but echocardiography showed a 15-18 mm echo-free space all around the heart and displayed a collapse sign of both atria during diastole. The cardiologist performed a pericardiocentesis because high risk of developing cardiac tamponade in the near future, which resulted in the rapid narrowing of the echo-free space, and a drainage tube was not placed. The pericardial fluid obtained was bloody. The patient was admitted to the hospital for observation, but no further accumulation of pericardial effusion was observed. However, within one to two weeks, the patient developed progressive bilateral pleural effusions and respiratory failure requiring supplemental oxygen and orthopnea. Two thoracentesis and pleural fluid analyses were performed, but no diagnosis was made. The patient’s worsening respiratory failure led to a referral to our hospital for further investigation.

**Figure 2:**
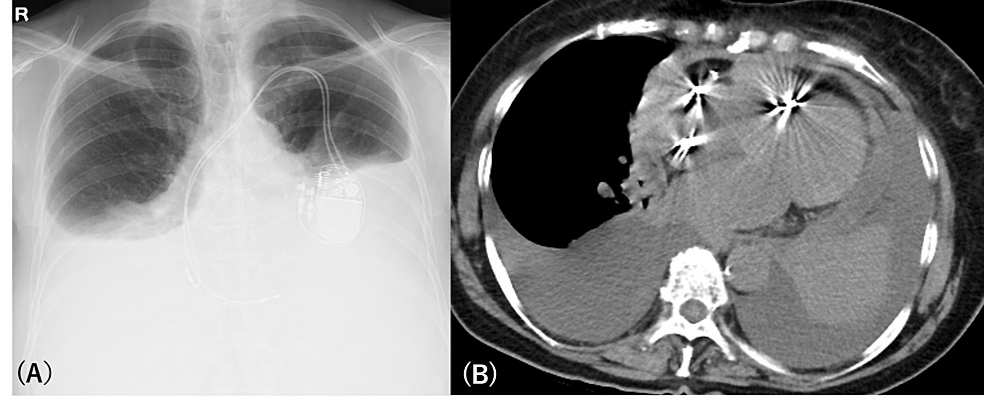
(A) Chest X-rays at the time of transfer to our hospital. (B) The axial view of CT of the chest showed small pericardial effusions and progressive bilateral pleural effusions at the time of transfer to our hospital

Upon admission to our healthcare facility, the patient was receiving supplemental oxygen through a nasal cannula at a rate of 2 L per minute, displaying a saturation level of 95%. Despite this, she was exhibiting tachypneic breathing at a rate of 29 breaths per minute while in the spine position. Even a minimal effort, such as being transferred to a wheelchair, resulted in a noticeable decline in oxygen saturation levels, dropping to a range between 70% and 80%. No signs of cardiac compromise were observed, including no distension of the jugular veins or peripheral edema. Chest X-ray and CT scans showed the presence of bilateral pleural effusion and a minor accumulation of pericardial fluid, but no infiltration shadow on the lungs or abnormalities in the abdomen and pelvis were identified (Figure 2A-2B).

**Figure 3:**
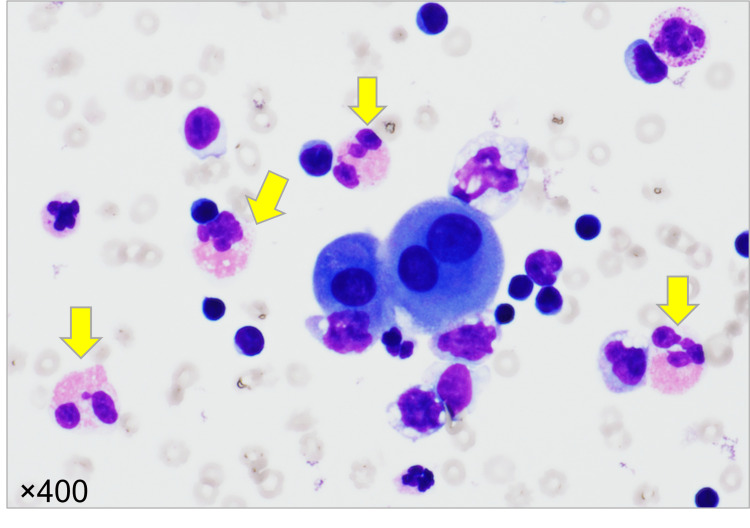
Gimesa-stained specimen of pleural effusion

Further examination of the pleural effusion at our hospital revealed it to be a serious fluid accumulation, and the results of the loop-mediated isothermal amplification method for tuberculosis were negative. The adenosine-deaminase level in the pleural fluid was also not elevated, and cytological analysis showed no malignant cells and only non-specific, primarily eosinophilic inflammatory findings consisting of a mixture of lymphocytes, histiocytes, granulocytes, and prominent eosinophils (Figure 3).

The patient’s complete blood count upon admission did not show elevated eosinophil levels, and biochemistry assessments indicated normal results for liver and renal function, electrolyte levels, serum protein, and albumin.

One week after admission, about one month after the onset of symptoms, the complete blood count showed an eosinophil count of 2051/µL, where the HES was first suspected. She was not taking any medications that could cause hypereosinophilia, and she had no history of staying or feeding in areas at high risk for parasitic infections. Screening for antibodies related to parasites, including Westermann’s and Miyazaki’s pulmonary aspiration, hepatolithiasis, hepatosuckinosis, Manson’s solitary worm, hookworm cysticercosiscanine filariasis, and others, showed no significant increase. Workup for myeloid neoplasm including bone marrow examination, tryptase, and vitamin B12 were normal. Although we were unable to search for abnormal T-cell clones by the flow cytometry method, it was concluded that lymphocyte-variant HES was unlikely due to normal thymus and activation-regulated chemokine (TARC) and serum immunoglobulin E levels. Thus, the final diagnosis was idiopathic HES. A summary of the test results used for differential diagnosis can be found in Table 1.

**Table 1:** Summary of investigations performed for differential diagnosis * Thymus and Activation-Regulated Chemokine, ** Antibody including Westermann’s and Miyazaki’s pulmonary aspiration, hepatolithiasis, hepatosuckinosis, Manson’s solitary worm, hookworm cysticercosiscanine filariasis, ***normal variants

Summary of investigations		
Biochmistry (pleural effusion)	Outcome	Reference
Total protein (g/dl)	4.1	Not available
Albumin (g/dl)	2.0	Not available
LDH (U/L)	197	Not available
ADA (U/L)	10.6	6.8-18.2
Hyaluronic acid (ng/ml)	4530	<30,000
Tuberculosis (LAMP method)	negative	negative
Biochemistry (serum)		
Total protein (g/dl)	6.6	6.6-8.1
Albumin (g/dl)	3.0	4.1-5.1
LDH (U/L)	199	124-222
Troponin I (ng/ml)	<0.01	0.0-0.02
BNP (pg/ml)	56.7	<18.4
Vitamin B12 (pg/ml)	878	197-771
Tryptase (ng/ml)	407	210-570
ACE-I (IU/L)	10.2	8.3-21.4
Lysozyme (µg/ml)	5.5	5.0-10.2
Immunology(serum)		
IgE (IU/mL)	226	<170
TRAC^*^ (pg/ml)	357	<450
IgG4 (mg/ml)	42.2	4.5-117
Antinuclear antibody (unit)	<40	<40
ANCA anti-MPO (U/ml)	negative	negative
ANCA anti-PR3 (U/ml)	negative	negative
Parasite Antibody Screening^**^	Class 0-1	Class 0-1
Bone marrow examination		
Karyotype	46XX, inv(9)(p12q13)^***^	46XX
4q12 (PDGFRA; FISH method)	0.0%	<2.0%

**Figure 4:**
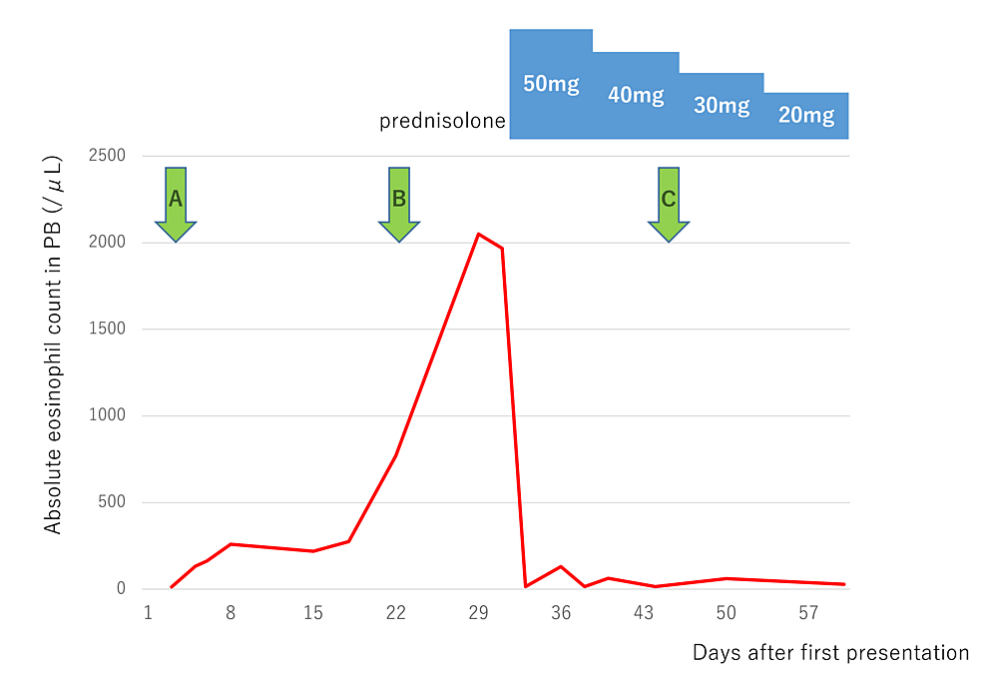
Peripheral blood eosinophil count A, at the initial visit to the previous doctor; B, at transfer to our hospital; C, at discharge

The treatment with prednisolone 1 mg/kg was initiated, leading to a swift decline in peripheral blood eosinophil counts (Figure 4).

**Figure 5:**
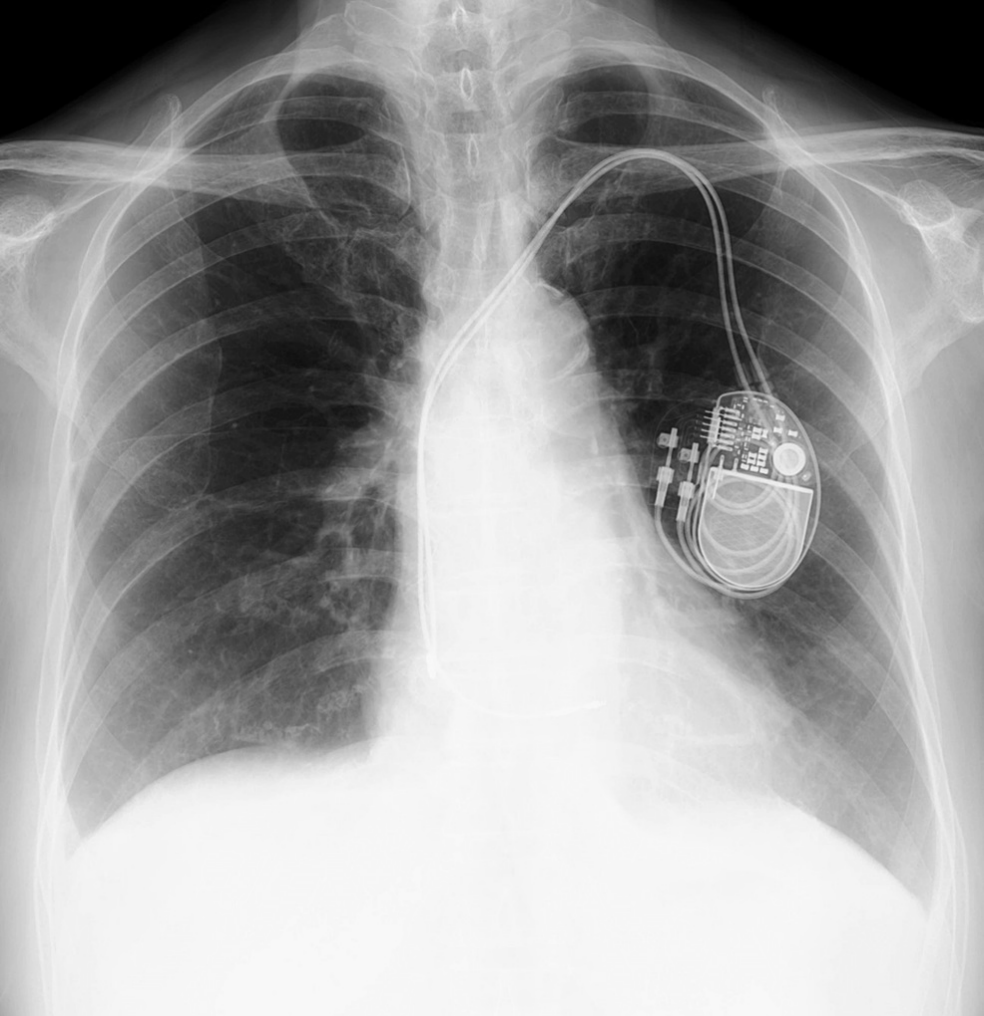
Chest X-rays on the 13th day of treatment with prednisolone

The bilateral pleural effusion and respiratory failure gradually improved as well, with a gradual tapering off of prednisolone every week. By the eighth day of treatment, oxygen supplementation was no longer required. On the 13th day, chest radiographs revealed a nearly complete absence of pleural and pericardial effusions (Figure 5).

The patient was discharged from the hospital approximately three weeks after the initiation of treatment. Upon being discharged from the hospital, the patient was required to attend the outpatient clinic for follow-up visits every two weeks. The purpose of these visits was to monitor their eosinophil counts, perform chest X-rays, and gradually decrease their prednisolone dosage. The treatment program was concluded about three months after it had started, and there has been no reoccurrence of symptoms. The patient will continue to receive regular follow-up appointments, ranging from every few months to every six months, to assess for any relapse of symptoms or signs of myocardial damage.

## Discussion

The presented case is of HES, which started with pericardial effusion and worsened with bilateral pleural effusion, leading to respiratory failure. The diagnosis was delayed for about a month, causing anxiety for the patient and family, as the progression of respiratory failure had not been effectively treated. The difficulties in diagnosis were due to the rarity of cardiac tamponade as the onset of symptoms of HES and a one-month time lag between the first symptoms and the appearance of hypereosinophilia in the peripheral blood.

The presence of subacutely progressive pericardial fluid and bilateral pleural effusions that were bloody and exudative pointed to the possibility of tuberculosis or malignancy. However, further examinations ruled out these possibilities. Other autoimmune diseases, such as systematic lupus erythematosus (SLE), and anti-neutrophil cytoplasmic antibody (ANCA)-associated vasculitis, were also considered as potential causes of multiple serositis. However, these were ruled out due to negative results in antinuclear antibody tests, the absence of granulomatous lesions suggestive of sarcoidosis on CT scans, and negative ANCA in myeloperoxidase or proteinase 3 results. One week after admission, about one month after the onset of symptoms, the complete blood count showed an eosinophil count of 2051/µL, where the HES was first suspected. After ruling out secondary hypereosinophilia medication, parasite, and collagen vasculitis, a search for myeloid neoplasms and those caused by abnormal lymphocyte clones were ruled out, and the disease was finally determined to be idiopathic hypereosinophilia.

There are no studies that have investigated the exact prevalence and incidence of HES in Japan. A retrospective analysis of the incidence in the US reported an estimated incidence of 0.315-6.3 per 100,000 [1], with most of the first-episode symptoms being cutaneous (37%), pulmonary (25%), or gastrointestinal (14%), and cardiac symptoms being reported in less than 5% of cases [2]. The pathophysiology underlying cardiac lesions in HES involves necrosis of myocardial cells caused by eosinophil infiltration, formation of endocardial thrombus leading to systemic thromboembolism, and fibrosis of the endocardium, as well as the mitral and tricuspid valves [3]. A case series that reviewed the existing literature reported that most of the cardiac lesions are left ventricular abnormalities, murmurs, and thrombi. Pericardial effusion (6/152; 3.9%) and pleural effusion (7/152; 4.2%) are infrequent symptoms in idiopathic HES [4]. However, it should be noted that the presence of publication bias cannot be ruled out. Cardiac tamponade without elevated troponin suggestive of myocardial damage, echocardiographic abnormalities of left ventricular abnormalities, or valvular disease, as in this case, is likely a rare phenotype of HES.

Furthermore, a key aspect of this case is that the peripheral blood eosinophil count was not elevated for a month after the onset of symptoms. HES is generally defined as an absolute eosinophil count of 1500/µL or higher and is associated with organ damage when seen repeatedly at intervals of one month or more. This definition implies that hypereosinophilia often precedes HES and leads to organ damage in the chronic course of the disease. In most reported cases presented rare pericarditis, the presence of hypereosinophilia in the peripheral blood at the time of initial diagnosis had been a trigger for suspicion [5-7]. In cases where organ damage precedes hypereosinophilia, it is challenging to suspect HES, but the presence of a large number of eosinophils among the inflammatory cells in the pleural effusion should raise suspicion.

## Conclusions

HES is a heterogeneous disease that causes various symptoms and can progress rapidly. It is possible that HES may be responsible for causing cardiac tamponade, although it is an uncommon occurrence. In certain instances of HES, manifestations of organ impairment occur prior to the elevation of peripheral blood eosinophilia. HES may be taken into account as a potential explanation for cases of inflammatory disease that result in unexplained damage, even if there is no clear indication of peripheral blood hypereosinophilia. Focusing on the type of inflammatory cells in the affected organ may help in the diagnosis.
